# Modulation of Circulating Cytokine-Chemokine Profile in Patients Affected by Chronic Venous Insufficiency Undergoing Surgical Hemodynamic Correction

**DOI:** 10.1155/2014/473765

**Published:** 2014-03-25

**Authors:** Veronica Tisato, Giorgio Zauli, Sergio Gianesini, Erica Menegatti, Laura Brunelli, Roberto Manfredini, Paolo Zamboni, Paola Secchiero

**Affiliations:** ^1^Department of Morphology, Surgery and Experimental Medicine and LTTA Centre, University of Ferrara, Via Fossato di Mortara 66, 44121 Ferrara, Italy; ^2^Institute for Maternal and Child Health, IRCCS “Burlo Garofolo”, Via dell'Istria 65/01, 34137 Trieste, Italy; ^3^Vascular Disease Center, University of Ferrara, Via Aldo Moro 8, 44124 Ferrara, Italy; ^4^Department of Medical Sciences, Operative Unit of Clinica Medica, University Hospital of Ferrara, Via Aldo Moro 8, 44124 Ferrara, Italy

## Abstract

The expression of proinflammatory cytokines/chemokines has been reported in *in vitro/ex vivo* settings of chronic venous insufficiency (CVI), but the identification of circulating mediators that might be associated with altered hemodynamic forces or might represent innovative biomarkers is still missing. In this study, the circulating levels of 31 cytokines/chemokines involved in inflammatory/angiogenic processes were analysed in (i) CVI patients at baseline before surgical hemody namic correction, (ii) healthy subjects, and (iii) CVI patients after surgery. In a subgroup of CVI patients, in whom the baseline levels of cytokines/chemokines were analyzed in paired blood samples obtained from varicose vein and forearm vein, EGF, PDGF, and RANTES were increased at the varicose vein site as compared to the general circulation. Moreover, while at baseline, CVI patients showed increased levels of 14 cytokines/chemokines as compared to healthy subjects, 6 months after surgery, 11 cytokines/chemokines levels were significantly reduced in the treated CVI patients as compared to the CVI patients before surgery. Of note, a patient who exhibited recurrence of the disease 6 months after surgery, showed higher levels of EGF, PDGF, and RANTES compared to nonrecurrent patients, highlighting the potential role of the EGF/PDGF/RANTES triad as sensitive biomarkers in the context of CVI.

## 1. Introduction

Chronic venous disease (CVD) is one of the most prevalent medical problems in the adult population of Western European countries and USA with a significant impact on afflicted patients and on the healthcare system [1]. Dysfunction of any of the normal structures of the venous system may lead to venous hypertension and to the development of chronic venous insufficiency (CVI), an advanced form of CVD presenting with a variety of signs and manifestations ranging from varicosities up to venous ulceration [2]. The available therapeutic options include conservative therapies (phlebotonic drugs, lower limbs elastic compression), ablative surgical or endovenous procedures (sclerotherapy, laser/thermal venous shrinkage) and saphenous sparing hemodynamic surgery [3]. Although a significant effort has been made on the development and evaluation of techniques that might assist in both diagnosis and treatment of CVI, the etiopathology of this condition still needs to be clarified and CVI remains a disease with a high recurrence rate that will be possibly reduced just whenever deeper comprehension of the pathophysiological mechanisms will be obtained [4, 5].

Clinical and basic science studies overall underlie that CVI results from a complex interplay of multiple factors, including the alteration of the hemodynamic forces acting on the vein wall that can be considered as a key event in CVI development [6, 7]. It is still unclear what initiates the inflammatory process in the vein wall, but it has been shown that the persistent venous hypertension characterizing CVI leads to an inflammatory response primarily mediated by leukocytes and involving cascades of cytokines/chemokines, matrix metalloproteinases activation, and alteration of endothelial cellular functions [8–11]. Expression of pro-inflammatory cytokines and chemokines has been reported in patients with varicose veins confirming the role of inflammation in CVI [12, 13]. In a recent work, we have shown a correlation between the PDGF-BB released by patient-derived vein endothelial cell (VEC) cultures and relevant hemodynamic parameters measured* in vivo* into the venous segments from which the VEC was isolated upon surgical ablation [14].

On these bases, to elucidate the link between systemic inflammation and altered hemodynamic forces, the primary aim of this study was to evaluate the effect of a saphenous sparing surgical correction (CHIVA strategy) [15] on the levels of circulating factors related to inflammation and angiogenesis characterizing CVI in order to identify a panel of biological markers able to correlate with the disease that might complement the standard procedures for diagnosis and posttreatment follow-up of CVI patients.

## 2. Materials and Methods

### 2.1. Patients and Samples Collection

The subjects involved in this study consisted of 32 patients (for a total of 60 plasma samples) affected by primary CVI with superficial venous reflux (C2-4EpAsPr following the CEAP classification) enrolled by the Vascular Disease Center at the University of Ferrara, in accordance with the Declaration of Helsinki and with approval obtained from the University-Hospital of Ferrara. All patients underwent a complete clinical assessment to evaluate the clinical, etiology, anatomy, and pathophysiology clinical class (CEAP) and to attribute a venous clinical severity score (VCSS) [7, 16]. At the same time, patients underwent echo-color-Doppler (ECD) scanning. The patient was examined in standing position with complete scanning of the great saphenous vein (GSV) and short saphenous vein (SSV) system, including junctions and tributaries. In addition, the main trunk of the deep venous system (external iliac, common, and superficial femoral veins, popliteal and gastrocnemius veins) and the perforators were completely examined. Calf muscular pump was elicited by manual squeezing, considering reflux the detection of a reverse flow longer than 0.5 sec in whatever of the above reported superficial and deep veins [17]. At the junction, level competence of the valve was also tested by the means of Valsalva manoeuvre as previously described [18]. Limitedly on the GSV, at 15 cm from the junction with the femoral vein, we also recorded, in addition to reflux time (RT), the following hemodynamic parameters: peak systolic velocity (PSV) and end diastolic velocity (EDV) that allowed the calculation of the resistance index (RI), as previously described [14]. After clinical assessment, patients underwent Ambulatory Conservative Hemodynamic Management of Varicose Veins (CHIVA) surgery, a hemodynamic minimally-invasive approach aiming to restore the venous drainage without eliminating the saphenous system [19, 20]. Blood samples were collected from patients before and/or after surgical treatment in the presence of sodium citrate. Samples were immediately centrifuged for plasma isolation that was stored at −80°C in single-use aliquots. For some patients (*n* = 6), before surgical treatment, paired blood samples were harvested from both the forearm vein and the more prominent and incompetent GSV tributary. Plasma samples obtained from blood harvested from the forearm vein of healthy subjects (age range: 25–60 years; gender: 60% men) were used as controls.

### 2.2. Analysis of Cytokines and Chemokines in Plasma Samples

Plasma samples were frozen and thawed only once before performing the MILLIPLEX MAP Human Cytokine/Chemokine Panel (Merck Millipore, Billerica, MA), a bead-based multiplex immunoassay, which allows the simultaneous quantification of the following 29 human cytokines: IL-1*α*, IL-1*β*, IL-1 receptor antagonist (ra), IL-2, IL-3, IL-4, IL-5, IL-6, IL-7, IL-8, IL-10, IL-12(p40), IL-12(p70), IL-13, IL-15, IL-17A, EGF, Eotaxin, G-CSF, GM-CSF, IFN-*α*2, IFN-*γ*, CXCL10, MCP-1, MIP-1*α*, MIP-1*β*, TNF-*α*, TNF-*β*, and VEGF. Moreover, a custom made MILLIPLEX MAP Human Cytokine/Chemokine Magnetic Bead Panel (Merck Millipore) was used to quantify the cytokines PDGF-AB/BB and RANTES (see Supplementary Table 1 available online at http://dx.doi.org/10.1155/2014/473765 for the list of the cytokines' abbreviations). Samples were processed in duplicate following the manufacturer's recommended protocols and read on a MAGPIX instrument equipped with the MILLIPLEX-Analyst Software using a five-parameter nonlinear regression formula to compute sample concentrations from the standard curves.

### 2.3. Network Analysis

Mapping of cytokines/chemokines into specific biological networks has been performed by using MetaCore analytical suite version 5.3 (GeneGo, MI, USA), an integrated analytical suite of algorithms based on a database of human protein-protein interactions, transcriptional factors, cell signaling, metabolism, and bioactive molecules linked to functional processes and diseases (http://www.genego.com/). Networks of cytokines/chemokines were created by using two different algorithms: (i) the network algorithm analysis generating subnetworks ranked by a *P* value and interpreted in terms of gene ontology processes to deduce top scoring processes that are regulated by differentially expressed proteins and (ii) the shortest paths algorithm to map the shortest path for interaction. The resulting networks were evaluated to determine which algorithm succeeded in creating modules that have higher than random saturation with the protein of interest. Networks were then graphically visualized as nodes (proteins) and edges (connection between proteins) alongside the pattern of expression.

### 2.4. Statistical Analysis

Data were calculated as median, mean ± standard deviation (SD). Box plots were used to show the median and interquartile values for each group of data. The results were evaluated by using analysis of variance with subsequent comparisons by Student's *t*-test and with the Mann-Whitney rank-sum test. Statistical significance was defined as *P* < 0.05.

## 3. Results

### 3.1. Study Population and Clinical Assessment

The main baseline demographic/clinical characteristics of the CVI patients are reported in Table 1. Overall, the 69% of enrolled patients were female with age ranging between 38 and 76 years and a mean age of 58.6 years, while male patients (31%) were characterized by age ranging between 37 and 61 years with a mean age of 50.3 years. At the baseline, assessment of VCSS ranged from 5 to 8 and corresponded to a CEAP clinical class ranging from C2 to C4. As far as the venous segments affected by CVI in our population, we found in all 32 cases the insufficiency of the GSV system, with reflux pattern involving also the SSV in 5 cases, the common femoral vein in 13 cases, the popliteal vein in 2 cases, and finally the gastrocnemius vein in 2 cases. Moreover, in our survey, the registered hemodynamic parameters at 15 cm from the sapheno-femoral junction were the following: peak systolic velocity (PSV) mean ± SD: 40.63 ± 14.05 cm/sec; end diastolic velocity (EDV), mean ± SD: −17.13 ± 7.95 cm/sec; resistance index (RI), mean ± SD: 1.43 ± 0.14; and reflux time (RT), mean ± SD: 2.97 ± 0.73 sec (Table 1).

### 3.2. The Levels of Selected Cytokines/Chemokines (RANTES, EGF, and PDGF) Are Higher in Varicose Vein Blood with respect to the Systemic Circulation

**Figure 1:**
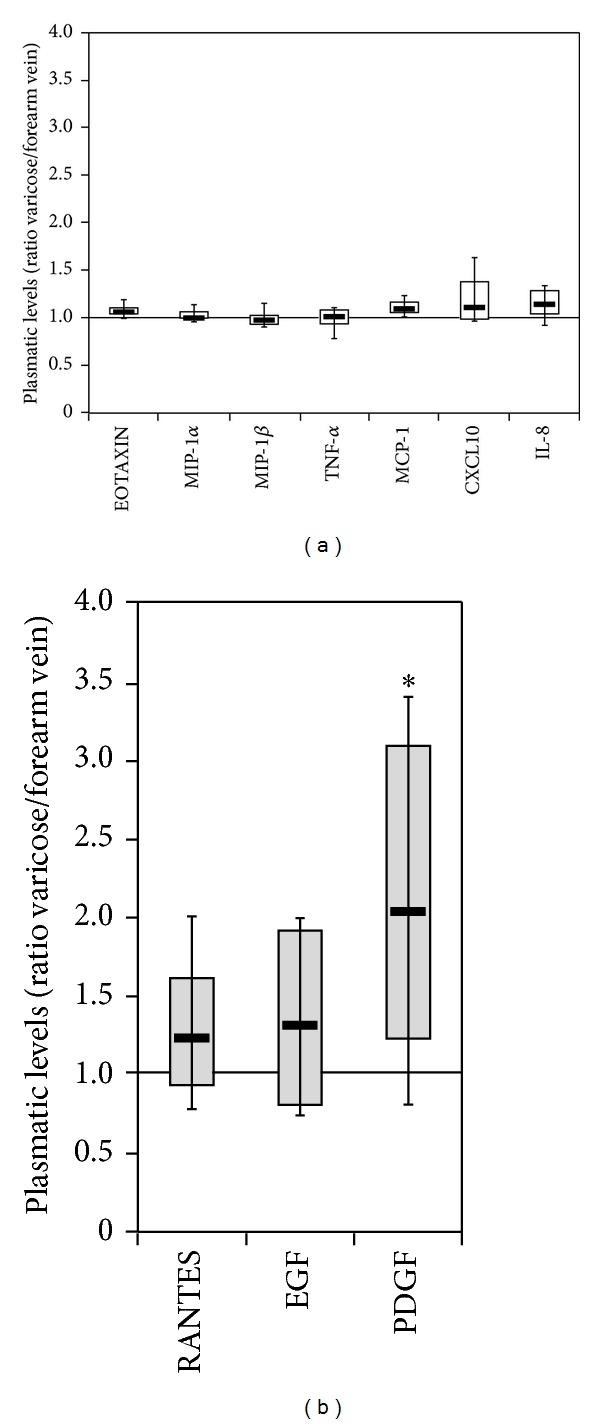
*Comparison between the levels of cytokines/chemokines measured in CVI patients in the varicose vein blood and in the systemic circulation*. The circulating levels of 31 cytokines/chemokines were measured in paired blood samples collected from the forearm and the varicose vein of CVI patients (*n* = 6). Data are expressed as ratio between local (varicose) and systemic (forearm) values. In (a), 7 representative cytokines/chemokines displaying the same levels at the varicose vein site and at systemic level are shown. In (b), the 3 cytokines displaying higher circulating levels (*P < 0.05) at the varicose vein site with respect to the systemic level are shown. Box plots are used to show the median and interquartile values for each group of data.

We have recently demonstrated that several cytokines/chemokines are significantly elevated in the plasma of patients affected by CVI with respect to healthy subjects, which suggested the presence of a systemic inflammation status in CVI patients [21]. In order to ascertain the contribution of local factors to the production and release of cytokines/chemokines found elevated in the general circulation, in a preliminary experiment, we have analyzed the levels of 31 cytokines/chemokines in paired blood samples collected both from the forearm vein and the varicose vein in a small number of patients (*n* = 6) (Figures 1(a)-1(b)). This pilot group included patients with a clinical score of 7 or 8 and C2 or C3 CEAP. Among the 31 cytokines/chemokines analyzed by multiplex assay, the following 18 were detectable, at different levels, in the plasma samples collected both from the arm and from the varicose vein in the leg: MIP-1*α*, IL-7, IL-8, IFN-*γ*, IL-12(p70), TNF-*α*, GM-CSF, IFN-*α*2, G-CSF, IL-1RA, MIP-1*β*, VEGF, EGF, EOTAXIN, MCP-1, CXCL10, PDGF AB/BB, and RANTES. The comparison between the levels measured in paired samples belonging to the same patient revealed the identity (with differences below 15%) between the systemic and varicose vein blood samples for most (15 out of 18) cytokines/chemokines, some of which are exemplified in Figure 1(a). For 3 cytokines/chemokines, we observed a mean increment between 20 and 35% for RANTES and EGF and a mean increment of 108 ± 24% for PDGF AB/BB (Figure 1(b)). This preliminary analysis demonstrates that the systemic profile matched the local profile for the great majority of cytokines and suggests that the increase of PDGF, RANTES, and EGF either contributes to the pathogenesis of the CVI or represents the response to the local vein damage.

### 3.3. Downmodulation of the Circulating Levels of Most Cytokines/Chemokines after Surgical Hemodynamic Correction

**Figure 2:**
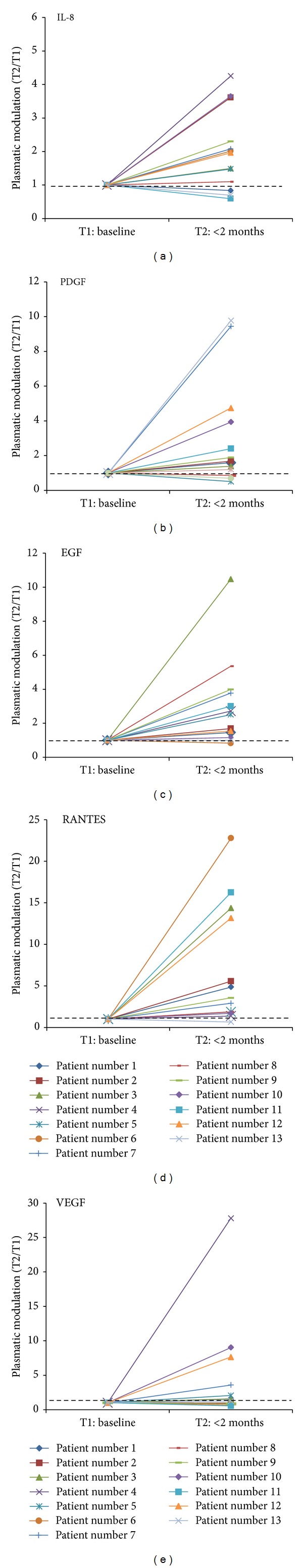
*Modulation of circulating cytokines/chemokines in CVI patients after <2 months of surgical hemodynamic correction.* The circulating levels of cytokines/chemokines were monitored in paired plasma samples of CVI patients (*n* = 13) before (at baseline, T1) and after (<2 months, T2) CHIVA. Data are shown as plasmatic modulation (T2/T1) for each patient. Hatched line indicates the baseline level set to 1.

All CVI patients enrolled in the study underwent CHIVA surgery, a conservative and minimally invasive therapeutic option for CVI aiming to restore the physiological lower limbs venous drainage without eliminating the saphenous system. Briefly, the functional restoration of the GSV was obtained once the incompetent GSV tributaries were flush ligated and/or once the reflux, which is coming from an incompetent saphenofemoral confluence, was suppressed by high ligation. In paired plasma samples harvested from forearm vein of CVI patients (*n* = 13) before and after (mean of 33 days) CHIVA, unexpectedly we documented a further significant increase (≥2 folds) with respect to presurgical levels for 5 cytokines: IL-8 (mean ± SD increase: 2 ± 1.2), PDGF (mean ± SD  increase: 3 ± 3.2), EGF (mean ± SD increase: 3 ± 2.6), RANTES (mean ± SD increase: 7 ± 7.2), and VEGF (mean ± SD increase: 4.4 ± 7.5) (Figure 2). Since these cytokines are known to play key roles in endothelial cell biology (in particular in promoting angiogenesis and endothelial cell remodeling), it is possible to suppose that their increase is a response to the surgical procedure. It is also noteworthy that 3 (EGF, PDGF, RANTES) out of 5 of these cytokines were those found elevated in the varicose vein as compared to the forearm vein plasma (Figure 1(b)).

**Figure 3:**
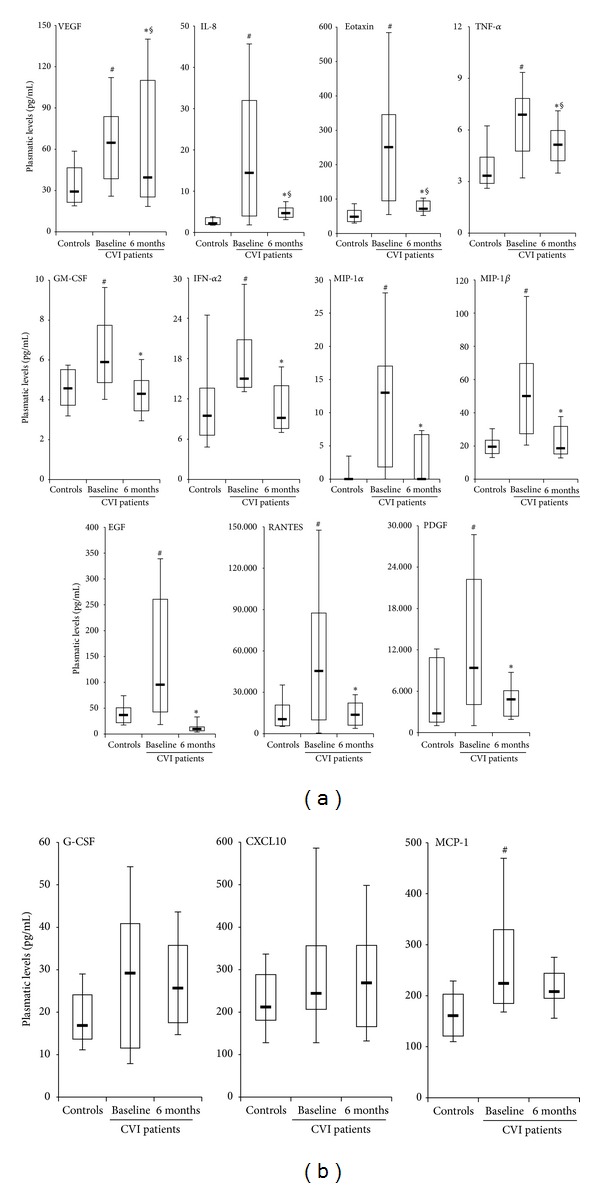
*Evaluation of circulating cytokines/chemokines in CVI patients 6 months after surgical hemodynamic correction.* The circulating levels of cytokines/chemokines were assessed in plasma samples harvested from forearm vein of healthy subjects (controls) and of CVI patients before (baseline) and after (6 months) CHIVA. In (a), the 11 cytokines/chemokines displaying lower levels after CHIVA, compared to baseline levels, are shown; *P < 0.05 compared to baseline; ^§^ *P* < 0.05 compared to controls. In (b), the 3 cytokines/chemokines displaying levels after CHIVA not significantly different compared to baseline levels are shown. In (a) and (b), horizontal bars are median, upper, and lower edges of box that are 75th and 25th percentiles, lines extending from box are 10th and 90th percentiles; ^#^ *P* < 0.05 compared to the controls.

**Figure 4:**
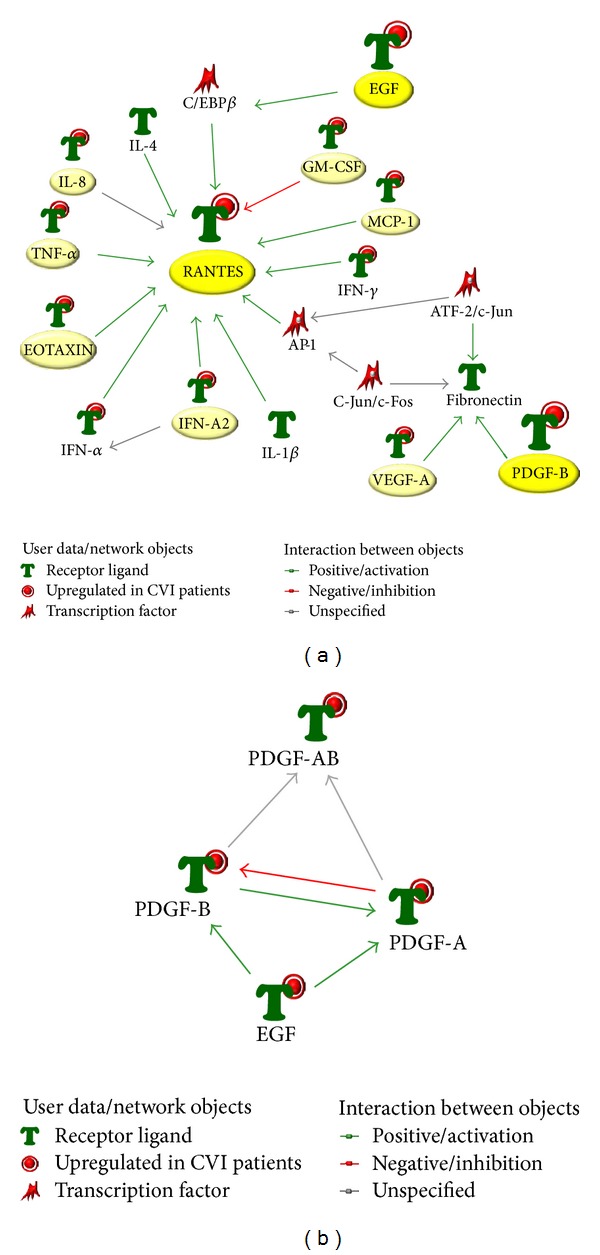
*Network analysis of the cytokines/chemokines differentially expressed in the context of CVI.* The identified panel of cytokines/chemokines characterizing CVI was assessed for network analysis. In (a), the top-score biological network generated by using the analysis network algorithm illustrating the connections among the differentially expressed cytokine/chemokine highlighting the role of RANTES as hub. In (b), proteins network between PDGF and EGF generated by the shortest path algorithm is shown.

However, the situation changed drastically when the cytokine and chemokine profile was analyzed in a group of patients (*n* = 9) 6 months after CHIVA and compared with the levels measured in healthy individuals and in pre-CHIVA patients (Figure 3). It should be underlined that 6 months after CHIVA, the clinical evaluation and the vascular parameters of the patients resulted significantly improved as reveled by (i) clinical score of 0 or 1, (ii) C0 or C1 CEAP clinical class, and (iii) hemodynamic parameters (RT, mean ± SD: 1.39 ± 0.53 sec; PSV, mean ± SD: 16.81 ± 6.51 cm/sec; EDV, mean ± SD: 8.31 ± 3.27 cm/sec) with the exception of one patient reporting a clinical score of 3 and C3 CEAP, with recurrent disease for failure of the surgical treatment. In line with the clinical observations, we found that the levels of 11 cytokines and chemokines were significantly (*P* < 0.05) lower with respect to the group of pre-surgical CVI patients, and for some cytokines/chemokines (including EGF, PDGF, and RANTES), the levels were not significantly different from the levels found in healthy controls (Figure 3(a)). Only three cytokines (G-CSF, CXCL10, and MCP-1) persisted at levels comparable to those measured in patients before CHIVA (Figure 3(b)). These data indicate that CHIVA is able to correct either completely or partially the circulating levels of several cytokines/chemokines altered in CVI. A network analysis of the cytokine pathways revealed the key role of RANTES as hub of the system (Figure 4(a)), with a strong direct interaction between PDGF and EGF (Figure 4(b)).

**Figure 5:**
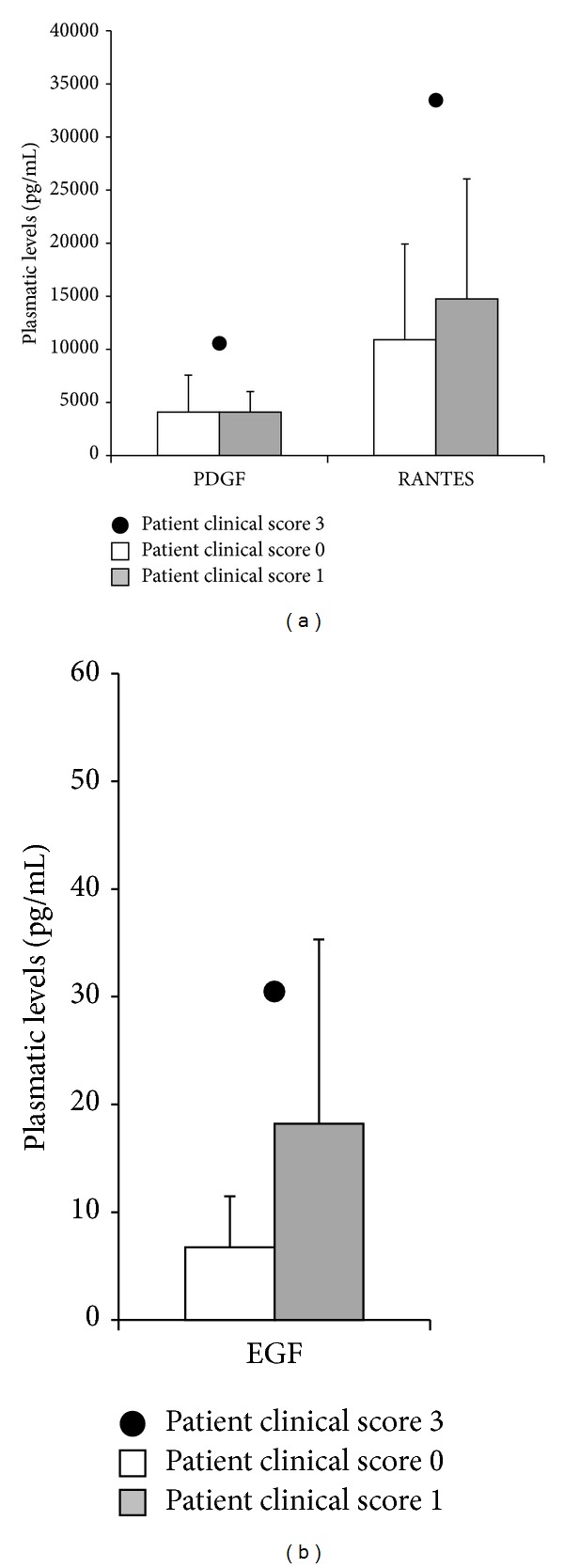
*Highest levels of PDGF, RANTES, and EGF in one CVI patient showing recurrence of the disease 6 months after CHIVA.* The plasmatic levels of PDGF, RANTES, and EGF assessed in the CVI-recurrent patient (clinical score 3) are shown in comparison to the means ± SD levels assessed in patients reporting clinical score 0 or 1.

In considering the potential link between cytokines/chemokines modulation and the clinical course after CHIVA, it is noteworthy that the only patient who exhibited a rapid clinical recurrence at 6 months of follow-up, as indicated by the clinical score 3, presented significantly higher levels (fold of increase >2) of EGF, PDGF, and RANTES with respect to the mean values measured in the patients with the best clinical parameters (clinical score 0-1) (Figure 5).

## 4. Discussion

The links between inflammation, endothelial activation/damage, and mechanical stretch/pressure in the context of CVI still need to be elucidated not only in terms of molecular mediators involved but also in terms of progression of the events. Incompetent valves with saphenous reflux (considered one of the most important hemodynamic factors in CVI) lead to endothelial activation and inflammation [2, 6–11], but there might be preexistent (inflammatory) conditions that might render the endothelium weaker and therefore more prone to lead to valve dysfunction. The effect of disturbed flow on vascular endothelium has been deeply evaluated* in vitro* in models of standard endothelium [11, 22–24] and, more recently, in an* ex vivo* model of primary venous endothelial cells (VEC) isolated from surgical saphenous specimens of CVI patients [12, 14]. Moreover, higher expression of proinflammatory cytokines/chemokines has been demonstrated at RNA level in varicose veins compared to control veins [13]. In further studies performed in the pathologic VEC model, we suggested the link between hemodynamic forces and the proinflammatory status of CVI, documented by a correlation between key hemodynamic parameters and the* ex  vivo* endothelial release of cytokines/chemokines, underlining in particular the possible contribution of soluble mediators such as PDGF-BB and osteoprotegerin [14, 25], a key regulator of the two TNF family members RANKL and TRAIL [26, 27]. Overall, these* in vitro/ex vivo* evidences support the hypothesis of the presence of circulating factors involved in the inflammatory/angiogenic processes related to CVI that are released (also, but perhaps not only) by the pathological endothelium that might be able to characterize the disease. In this light, we here demonstrate that, within the plasmatic cytokines/chemokines upregulated in CVI patients compared to healthy controls, the circulating levels of selected factors (including IL-8, PDGF, EGF, VEGF, and RANTES) were further increased in a short time follow-up (<2 months) after hemodynamic correction by CHIVA, perhaps as direct consequence of the surgical procedure since they are all factors involved in endothelial remodeling. On the contrary, in patients assessed 6 months after CHIVA, with the exception of MCP-1, G-CSF, and CXCL10, we documented a significant decrease of the cytokines/chemokines, returning, in some cases, to levels similar to healthy individuals. These observations prove that the hemodynamic correction is able to restore the normal levels of several inflammatory/angiogenic factors characterizing CVI. Of interest, in a patient that at the 6 months after CHIVA presented a relapse of the disease (clinical score 3), we observed higher levels of EGF, PDGF, and RANTES, with respect to the patients after CHIVA characterized by clinical score 0-1. It is important to underline that the findings reported in the present study have been obtained comparing healthy subjects and groups of CVI patients monitored at the same season (winter) since in preliminary analysis, we observed a “season-dependent” effect on the circulating levels of the considered cytokines/chemokines, in agreement with previous studies [28–30].

We are aware about the limitations of this pilot study, mainly due to the low number of subjects and the short times of the postsurgery follow-up of CVI patients due to the seasonal limitation for a proper comparison of the cytokines/chemokines levels. However, our results suggest a significant link between* in vivo* hemodynamic forces and increased circulating levels of inflammatory/angiogenic mediators (including EGF, PDGF, and RANTES). Although these circulating factors likely represent the response to CVI inflammation, we cannot exclude their contribution to the pathogenesis of varicose veins. Nonetheless, we here propose the potential role of a panel of selected cytokines, including EGF, PDGF, and RANTES, as biomarkers in the characterization of the disease progression and in the postsurgery monitoring of patients to predict disease recurrence (once their levels increase). This hypothesis is furthermore supported by the evidence that, even though we have demonstrated that the systemic profile matched the local profile, these three molecules showed the major levels at the varicose vein site. Of note, since RANTES acts as chemotactic molecule for leukocytes [13, 31], it actually appears to represent one of the hubs in the cytokine network (Figure 4(a)) and a link between the endothelial disease and the inflammatory process.

These preliminary observations are therefore the proof of concept for future clinical studies involving a larger cohort of patients with the aim of identifying CVI-specific circulating biomarkers to be used in the clinical practice at the diagnostic level and during the post-treatment follow-up of CVI patients.

## Supplementary Material

Supplementary Table: Panel of Cytokines and growth factors analyzed by multiplex immunoassay in patients' samples.

## Figures and Tables

**Table 1:** Baseline demographic/clinical characteristics of the CVI patients (*n* = 32).

	%	Mean ± SD	Range
Gender	—		
Men/women	31/69	—	—
Patient age (years)			
Men	—	50.3 ± 12.5	37/61
Women	—	58.6 ± 12.5	38/76
CEAP classification			
C2	37	—	—
C3	44	—	—
C4	19	—	—
Primary etiology	100	—	—
Superficial venous reflux	100	—	—
Reflux only	100	—	—
Hemodynamic parameters			
Peak systolic velocity (cm/sec)	—	40.63 ± 14.05	15/68
End diastolic velocity (cm/sec)	—	−17.13 ± 7.95	−35/−4
Resistance index	—	1.43 ± 0.14	1.19/1.74
Reflux time (sec)	—	2.97 ± 0.73	1.70/4.60
Clinical score (VCSS)			
5	6	—	—
6	28	—	—
7	38	—	—
8	28	—	—
