# Supplementary material for: Modulation of Circulating Cytokine-Chemokine Profile in Patients Affected by Chronic Venous Insufficiency Undergoing Surgical Hemodynamic Correction
Source: J Immunol Res. 2014 Mar 25;2014:473765. doi: 10.1155/2014/473765 (PMC3984831; doi:10.1155/2014/473765)
Supplement: Supplementary file 1 — Supplementary Table: Panel of Cytokines and growth factors analyzed by multiplex immunoassay in patients' samples. [file 473765.f1.pdf]

SUPPLEMENTARY MATERIAL

Supplementary Table 1. Cytokines and growth factors analyzed by multiplex immunoassay

**IL-1α**	Interleukin 1, alpha
**IL-1β**	Interleukin 1, beta
**IL-1ra**	Interleukin-1 receptor antagonist
**IL-2**	Interleukin 2, TCGF, lymphokine
**IL-3**	Interleukin 3, colony-stimulating factor, MCGF; MULTI-CSF
**IL-4**	Interleukin 4, B cell growth factor 1
**IL-5**	Interleukin 5, B-cell differentiation factor I; T-cell replacing factor
**IL-6**	Interleukin 6, B-cell differentiation factor; B-cell stimulatory factor 2
**IL-7**	Interleukin-7
**IL-8**	Interleukin 8, CXCL8, alveolar macrophage chemotactic factor I
**IL-10**	Interleukin 10, T-cell growth inhibitory factor
**IL-12 (p40)**	Interleukin-12 subunit p40,
**IL-12 (p70)**	Interleukin-12 subunit p70, CLMF, NKSF
**IL-13**	Interleukin 13, ALRH, BHR1
**IL-15**	Interleukin 15
**IL-17A**	Interleukin 17, CTLA-8; cytotoxic T-lymphocyte-associated antigen 8
**EGF**	Epidermal growth factor
**Eotaxin**	C-C motif chemokine 11; eosinophil chemotactic protein
**G-CSF**	Granulocyte colony-stimulating factor
**GM-CSF**	Granulocyte-Macrophage Colony Stimulating Factor
**IFN-α2**	Interferon alpha 2
**IFN-γ**	Interferon gamma
**CXCL10**	IP10, 10 kDa interferon gamma-induced protein
**MCP-1**	CCL2, C-C motif chemokine 2; monocyte chemoattractant protein 1
**MIP-1α**	CCL3, C-C motif chemokine 3; G0/G1 switch regulatory protein 19-1
**PDGF**	Platelet derived growth factor
**MIP-1β**	CCL4, C-C motif chemokine 4; CC chemokine ligand 4
**RANTES**	CCL5, C-C motif chemokine 5; SIS-delta
**TNF-α**	Tumor necrosis factor, alpha
**TNF-β**	Tumor necrosis factor, beta
**VEGF**	Vascular endothelial growth factor
